# The quest for modernisation of traditional Chinese medicine

**DOI:** 10.1186/1472-6882-13-132

**Published:** 2013-06-13

**Authors:** Qihe Xu, Rudolf Bauer, Bruce M Hendry, Tai-Ping Fan, Zhongzhen Zhao, Pierre Duez, Monique SJ Simmonds, Claudia M Witt, Aiping Lu, Nicola Robinson, De-an Guo, Peter J Hylands

**Affiliations:** 1 King's College London, Department of Renal Medicine, London, UK; 2 Institute of Pharmaceutical Sciences, Department of Pharmacognosy, Karl-Franzens-University Graz, Graz, Austria; 3 Department of Pharmacology, University of Cambridge, Cambridge, UK; 4 School of Chinese Medicine, Hong Kong Baptist University, Hong Kong, China; 5 Laboratory of Pharmacognosy, Bromatology and Human Nutrition, Université Libre de Bruxelles, Brussels, Belgium; 6 Royal Botanic Gardens, Kew, Richmond, Surrey, UK; 7 Institute for Social Medicine, Epidemiology and Health Economics, Charité-Universitätsmedizin, Berlin, Germany; 8 Allied Health Sciences, Faculty of Health and Social Care, London South Bank University, London, UK; 9 Shanghai Research Centre for TCM Modernisation, Shanghai Institute of Materia Medica, Chinese Academy of Sciences, Shanghai, China; 10 King's College London, Institute of Pharmaceutical Science, London, UK

**Keywords:** Evidence-based medicine, Chinese herbal medicine, Acupuncture, History, Science, Efficacy, Safety, Integrity, Integration, Innovation

## Abstract

Traditional Chinese medicine (TCM) is an integral part of mainstream medicine in China. Due to its worldwide use, potential impact on healthcare and opportunities for new drug development, TCM is also of great international interest. Recently, a new era for modernisation of TCM was launched with the successful completion of the *Good Practice in Traditional Chinese Medicine Research in the Post-genomic Era* (GP-TCM) project, the European Union’s Seventh Framework Programme (FP7) coordination action on TCM research. This 3.5-year project that involved inputs from over 200 scientists resulted in the production of 20 editorials and in-depth reviews on different aspects of TCM that were published in a special issue of *Journal of Ethnopharmacology* (2012; volume 140, issue 3). In this narrative review, we aim to summarise the findings of the FP7 GP-TCM project and highlight the relevance of TCM to modern medicine within a historical and international context. Advances in TCM research since the 1950s can be characterised into three phases: Phase I (1950s-1970s) was fundamental for developing TCM higher education, research and hospital networks in China; Phase II (1980s-2000s) was critical for developing legal, economic and scientific foundations and international networks for TCM; and Phase III (2011 onwards) is concentrating on consolidating the scientific basis and clinical practice of TCM through interdisciplinary, interregional and intersectoral collaborations. Taking into account the quality and safety requirements newly imposed by a globalised market, we especially highlight the scientific evidence behind TCM, update the most important milestones and pitfalls, and propose *integrity*, *integration* and *innovation* as key principles for further modernisation of TCM. These principles will serve as foundations for further research and development of TCM, and for its future integration into tomorrow’s medicine.

## Introduction

Traditional Chinese medicine (TCM) is a holistic medical system for diagnosis, prevention and treatment of diseases and has been an integral part of Asian cultures for thousands of years. TCM uses experience-based therapies such as acupuncture and herbal medicine and is characterised by its underpinning theoretical guide, i.e. the philosophy of Yin-Yang balance [1-5]. In a historical and international perspective, this report discusses modernisation of TCM, an effort to bring the ancient practice of TCM in line with modern scientific standards [6]. Focusing on the past 60 years since the 1950s, we aim to highlight the scientific evidence behind TCM, update the most important milestones and pitfalls, and propose key principles to guide future developments. Due to the wide range of information needed for this narrative review, inclusion and exclusion of literature were judged by co-authors who are experts in the related research area, based on the quality of work but also on the necessity for illustrating specific research areas. Consensus was sought among all authors if quality and credibility of any sources of information were questioned by any co-author.

## Review

These past 60 years can be divided into two 30-year phases, which are followed by a prospective phase III that will be crucial for the scientific prospects of TCM.

• Phase I (1950s-1970s): developing TCM higher education, research and hospital networks in China;

• Phase II (1980s-2000s): developing legal, economic & scientific foundations and international networks for TCM; and

• Phase III (2011 onwards): further consolidating the scientific basis and clinical practice of TCM.

### Phase I (1950s-1970s)

In response to the open invitation from Premier Zhou Enlai to “come and see” the New China in the early 1950s, a British Labour Party delegation visited China in 1954 [7]. Upon its return, Professor Derrick B. James, the then Lecturer in Anatomy at University College London, contributed a report to *the Lancet* under the theme of *The Wider World* to introduce the ambition of the New China to modernise TCM [8]. Historically, this seminal article served as an international harbinger of the TCM modernisation campaign, saying,

“[the] *hypothesis is not that Chinese medicine is anachronistic, but that its claims are worth investigation, so that anything useful it contains may be incorporated into the body of modern Chinese medical practice*” [8].

The 1950s were the beginning of China’s efforts to modernise TCM. It was not until then that China started to build its national networks of TCM universities, hospitals and research institutes, which laid the structural foundation for modernisation of TCM. Importantly, China’s ten most prestigious TCM universities and its national TCM academy were all founded between 1954 and 1960. By the early 1980s, 25 TCM colleges had been established and the number of beds in TCM hospitals increased by 28.5-fold from 84,625 in 1949 to 2,412,362 in 1984 [1].

With the exception of a few Western scientists like Manfred Porkert, who studied TCM in China and brought it to the West [9], this phase was largely closed to the West due to the Great Leap Forward, the Great Cultural Revolution and the Cold War. Nevertheless, these thirty years had fundamental and lasting effects on the modernisation of TCM.

• Collation of case reports and sharing of experience within the TCM community, provision of TCM knowledge in the curriculum of conventional medical colleges in China and teaching of modern medicine in TCM colleges paved the way for further development of TCM, as well as mutual understanding and integration of the two medical traditions [10];

• The effect of acupuncture on pain was studied, although its dependence on specific acupuncture points was not proven [11]. In addition, the analgesic effect of acupuncture was shown to be antagonised by the opioid antagonist naloxone in both mice and humans [12,13], suggesting that endogenous narcotic(s) might mediate the effect of acupuncture, as confirmed later by Han [14]; and

• Many ground-breaking TCM-inspired discoveries date back to this period of time, including the award-winning discoveries of the anti-malaria drug artemisinin from the Chinese plant *Artemisia annua* L. by Youyou Tu and co-workers [15-17], the isolation of the anti-cancer compound camptothecin from *Camptotheca acuminata* Decne, by Monroe Wall, Mansukh Wani and co-workers [18,19]. Also arsenic trioxide was discovered during this time as a new adjuvant treatment for leukaemia [20].

### Phase II (1980s-2000s)

This phase was especially characterised by the following main hallmarks:

• In 1985, China’s State Administration of Traditional Chinese Medicine (SATCM) was established and it has since coordinated actions nationally and played an active role internationally on TCM-related matters;

• In 1998, the American Chemical Society granted Monroe Wall the Alfred Burger Award, the most prestigious prize in medicinal chemistry and, in 2000, he and Mansukh Wani won the Charles F. Kettering Prize, an international award, from the General Motors Foundation for the discovery of camptothecin and taxol, two ground-breaking natural products with anti-cancer activity [21]; and

• In 2003, China's National Science & Technology Progress Award honoured Keji Chen, Lianda Li and colleagues in the China Academy of Chinese Medical Sciences with a top category award for their successes in TCM treatment of cardiovascular diseases.

During this thirty-year phase, China’s national TCM network continued to grow, along with international outreach, and to integrate with Western medicine. The latter has been coordinated by the Chinese Association of Integrative Medicine since late 1981. By the 1990s, China had almost 3,000 dedicated TCM hospitals and over 95% of Western medicine hospitals in China had fully-fledged TCM wards and outpatient departments; outside China, TCM was practised by more than 300,000 practitioners in over 140 countries [3]. In 1991, the first university related TCM hospital was opened in Koetzting, Germany, as a joint venture with Dongzhimen Hospital, Beijing [22]. Since then, numerous TCM hospitals, clinics and teaching programmes have been established in Europe and other parts of the world [3].

As China’s gross domestic product (GDP) increased from No. 7–10 in the world at the beginning of the 1980s to No. 2 in 2010, it continued to strengthen its national TCM teaching and training capacity. By the end of the 2000s, China had 32 higher education institutions (HEIs) specialising in TCM and additional 52 HEIs with TCM majors; some 270,000 students were in training at all levels [23]. Spearheaded by the Ministry of Science and Technology, the Ministry of Health and SATCM, and with support from 16 ministries, China has continuously increased its investment in the R&D of TCM and promoted international dialogues and collaborations, especially since the late 2000s [6].

Meanwhile, funding bodies outside China also started to increase their support for research on non-conventional medicines, such as TCM. In 1998, the US National Institutes of Health established the National Center for Complementary and Alternative Medicine (NCCAM), dedicated to funding research on complementary and alternative medicines, including TCM [24]. With a starting annual budget of 50 million dollars for 1999, the Center’s annual funding has been in the region of 100–125 million dollars in the past ten years. In 2007, the Australian National Institute of Complementary Medicine (NICM) was established following the 2003 recommendations by the Expert Committee on Complementary Medicines in the Australian Health System. This was to provide leadership and support for strategically directed research into complementary medicine and translation of evidence into clinical practice and health policy. Initial seed funding for the establishment of NICM in 2007 included 4 million Australian dollars from the Department of Health and Ageing and 0.6 million Australian dollars from the New South Wales Office for Science and Medical Research [25]. In 2005, Austria-based Eurasia-Pacific Uninet and the China Academy of Chinese Medical Sciences signed the first Memorandum of Agreement on Sino-Austrian collaboration in TCM research. Following this in 2007, the Sino-Austrian Collaborating Centre for Chinese Medical Sciences was established in Beijing, with a joint project on TCM and age-related diseases funded with one million Euro from both the Austrian Federal Ministries of Health and of Science and Research, together with the China Ministry of Science and Technology [26]. In 2009, the European Union (EU) funded ~1 million Euro to the *Good Practice in Traditional Chinese Medicine (GP-TCM) Research in the Post-genomic Era* Consortium under its 7^th^ Framework Programme (FP7), in order to coordinate EU-China dialogues and collaborations in TCM research [27]. International collaborations in TCM practice, teaching, research and development were also promoted by new international organisations established in the five years since 2000, e.g. the International Society for Chinese Medicine, the Modernized Chinese Medicine International Association, the Consortium for Globalization of Chinese Medicine and the World Federation of Chinese Medicine Societies, each with distinct strengths [27]. Existing international organisations also took actions. The World Health Organization (WHO) recognised the importance of TCM, and published *Medicinal Plants in China: A Selection of 150 Commonly Used Species* in 1989 [28]. In 2010 it set up a programme to standardise terms used in TCM and its derivatives such as *kampo* (Japanese herbal medicines derived from TCM), as the beginning of its efforts to create an evidence base for traditional medicine and to provide an international platform to harmonise information exchange on traditional medicines [29]. The International Organization for Standardization (ISO) set up specialised committees dedicated to TCM in 2009 [30]. The United Nations Educational, Scientific and Cultural Organization (UNESCO) inscribed acupuncture and moxibustion on the *Representative List of the Intangible Cultural Heritage of Humanity* in 2010 [31] and inscribed *Ben Cao Gang Mu* (Compendium of Materia Medica) and *Huang Di Nei Jing* (Yellow Emperor's Inner Canon) in the Memory of the World Register one year later [32,33].

Over the years, international journals and public media sensed the historic opportunities and challenges in TCM. In *the Lancet*, for example, a number of aspects of TCM, such as acupuncture [34-36], TCM diagnostic methods [2,37], herb-drug and herb-immunoassay interactions [38,39], as well as therapeutic and sometimes severe adverse effects of TCM drugs and adulterants [40-44] were reported. In recognition of the political, historic and cultural impacts on the development of TCM [4,5,45,46], *the Lancet* highlighted the development of TCM in different countries and regions, characterised by global perspectives [2,37,42,47-49].

Governmental and non-governmental efforts within and outside China have coincided with new breakthroughs in modern science. Especially during the past two decades, globalisation, digitisation, the use of the internet, “omics” technologies and the concept of systems biology have provided unprecedented opportunities for international collaborations and provision of new strategies and powerful tools for revisiting TCM scientifically. Representative examples to illustrate the main achievements of these thirty years with international and scientific perspectives are given below.

#### TCM resources

**Figure 1:**
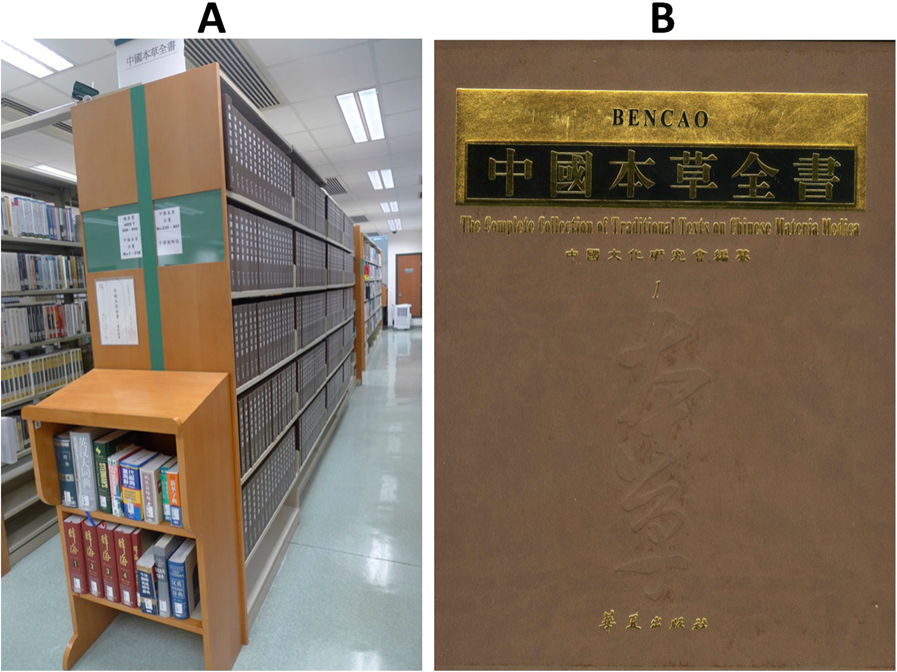
**TCM has continuous records of thousands of years. A**. *The Complete Collection of Traditional Texts on Chinese Materia Medica* in the Hong Kong Baptist University Library; **B**. Cover image of the volume one. Photos were taken in November 2011 by Professor Zhongzhen Zhao, School of Chinese Medicine, Hong Kong Baptist University, China.

From 1983 to 1994, China conducted a series of nationwide surveys on the substances used in TCM practice across the country. According to the official data published by the SATCM, 11,146 botanical and 1,581 zoological species, as well as 80 minerals were used [50]. In 2000, the *Complete Collection of Traditional Texts on Chinese Materia Medica* was published by the Hua Xia Publishing House, Beijing, China (Figure 1). In 410 volumes and more than 246,000 pages, more than 800 classic monographs are included, making it the most comprehensive compilation of TCM and Chinese materia medica classics dating back to 220 BC - 1911 AD. This collection not only highlights the value of TCM as a rich source for knowledge-based medical rediscovery due to its continuous documentation of clinical experiences for thousands of years, but also implies the huge task to dissect out the very best parts of TCM for modern innovation. The recognition of the need to standardise materials used in TCM is evidenced in the nine editions of the Chinese Pharmacopoeia, with each edition containing updated information about the medicinal products used in TCM. In its latest edition published in 2010, Volume I is fully dedicated to TCM drugs, representing the most comprehensive official monographs in this field. Peigen Xiao, the founding director of the Institute of Medicinal Plant Development in Beijing, was a pioneer in systematic studies of TCM resources, especially Chinese medicinal plants. He built bridges between Chinese and Western pharmacognosy and introduced Chinese medicinal plants to the rest of the world. His work resulted in the discovery of new species and the publication of hundreds of scientific papers and more than 25 books, e.g. [51-54]. In response to the European use of TCM products, Hildebert Wagner, Rudolf Bauer and Peigen Xiao started a joint project in 1995 to elaborate chemical fingerprint analytical methods for the identification of Chinese herbs in Western pharmacies, leading to the publication of two volumes of books [55]. In 2008, the European Pharmacopoeia Commission set up a specialised committee dedicated to TCM and started to draft monographs on TCM herbs [56]. The US Pharmacopoeia has also included some Chinese herbs in their monographs.

#### Efficacy and effectiveness of Chinese herbal medicine

In the 1990s, randomised, controlled clinical trials began to successfully investigate claims for a series of TCM drugs. Atherton and colleagues conducted a series of such trials in the UK, investigating the efficacy of a TCM formula in the treatment of atopic eczema [41,57]. In Japan, double-blind placebo-controlled trials showed two *kampo* drugs to be both safe and efficacious in the treatment of constipation and perennial nasal allergy [48]. In Australia, two TCM products were shown to be efficacious in the treatment of irritable bowel syndrome and hepatitis C [58,59]. These clinical trials, among others, set important milestones for the overseas use of Chinese herbal medicine. In 2003, Kanglaite, a botanical drug derived from *Coix lacryma-jobi* L. seeds became the first drug derived from a TCM herbal remedy to go into clinical trials in the USA [60]. Subsequently, many TCM-derived products developed in China (such as Dantonic® for chronic stable angina pectoris and Fuzhenghuayu® for liver fibrosis) or the USA (PHY906® as an adjuvant cancer remedy) have been approved for clinical trials by the US Food and Drug Administration (FDA). In 2010 it was estimated that approximately 25% of botanical investigational new drug (IND) applications submitted to the FDA were derived from TCM herbs [61]. On the other hand, according to Cochrane and other independent reviews, many clinical trials on TCM were inconclusive, at least partially because of the low quality of many trials conducted in China [62]. Similarly, the quality of systematic reviews and data meta-analysis has also been criticised [63]. This situation is about to change. Since the mid-2000s, China has accelerated its steps in joining the international community of evidence-based medicine and clinical trials conducted in China have begun to be published in leading clinical journals [49,64,65]. Nevertheless, many challenges remain [43]. Some challenges are specific to TCM including the practice of personalised medicine and the difficulties in designing quality controls for TCM practices and drugs, about which international collaborations such as GP-TCM have led to the development of practical guidelines [66,67].

#### Adverse effects of Chinese herbal medicine

By contrast with the highly regulated field of Western drugs, for which safety is a paramount preoccupation, the many centuries of traditional use of so-called “natural products” is, for the general public, often erroneously believed to be innocuous because they are “natural”. Traditional use, however, is only an indication but certainly not a proof of safety as dreadful mid-term and long-term toxicities have a low chance of being detected [68]. This is being increasingly recognised since 1993 when Vanherweghem and colleagues reported rapidly progressive interstitial renal fibrosis in women who had followed a slimming regimen inadvertently containing *Aristolochia sp* roots. Aristolochic acids were identified as mediators of not only the *Aristolochia*-induced renal fibrosis, the endemic Balkan nephropathy, but also *Aristolochia*-induced urothelial cancer [69,70]. In 1995, the Victorian Department of Human Services and the Health Departments of New South Wales and Queensland in Australia commenced a review in order to assess the risks and benefits associated with the practice of TCM [71]. These studies, together with some other reports, such as those on herb-drug interactions [38], aconite poisoning [44] and the hepato- and genotoxicity of pyrrolizidine alkaloids [72], gave an impetus to research the adverse effects of herbal medicines. Given the number of herbal products on the market and relatively low budgets available for research so far, safety assessment has been carried out on relatively few herbs according to modern guidelines [68]. This emphasises the importance of pharmacovigilance in detecting any unfortunate adverse events [73,74].

#### Legislation and regulation of Chinese herbal medicine

Given the importance of ensuring quality of TCM drugs and safety of patients, legislation and regulation of TCM practitioners and products are equally important. In particular, the approval of a green tea extract (Veregen®) as a drug in the USA and some European countries indicates that, although “new” botanical drugs pose many challenges for both industry and regulatory agencies, standardisation and stringent regulation of these complex drugs are achievable [75]. In the EU, the Committee on Herbal Medicinal Products (HMPC) of the European Medicines Agency (EMA) regulates the use of herbals and publishes herbal monographs and lists, which present a documented scientific opinion on the safety and efficacy data of herbal substances and their preparations intended for medicinal use [76]. Specifically, the HMPC evaluates scientifically all available information including non-clinical and clinical data but also documents long-standing use and experience in the EU. Due to the different approaches from those used by the FDA, the European HMPC adopted practical and relevant guidelines, which have brought back hundreds of herbals to the pharmaceutical standards of quality as approved drugs, under the “well-established” and “traditional” registration schemes, while the FDA approved so far only a few. Dobos and co-workers gave an overview of the status of regulations in the USA, the UK, Germany, Australia and China in 2005 [77]. The different approaches of regulating herbal products have been also recognised as an important issue by the FP7 GP-TCM Consortium, which compiled a more comprehensive global comparison of herbal regulation practices in order to promote harmonisation in the future [61].

#### Quality control and standardisation of Chinese herbal medicine

For the future development of TCM, raw materials must be produced in a sustainable way. Cultivation under good agricultural practice (GAP) should be the goal [78,79]. The scientific principles of *daodi* should be also considered – *Daodi* medicinal materials are those produced and assembled in specific geographic regions with designated natural conditions and ecological environment, with particular attention to cultivation technique, harvesting and processing, and thus regarded as superior in quality and effects as compared to materials of the same species obtained from other regions [80]. The identity and pharmaceutical quality of herbal medicinal products must be controlled in Europe and China according to the pharmacopoeia standards [81]. Due to the complex chemical composition of TCM drugs and many sources of variations that could lead to batch-to-batch inconsistency, classical quality control measures such as those applied to purified chemical drugs are often not suited to ensure quality of TCM materials and products. Sometimes DNA based methods may be needed for unambiguous authentication [82], but do not yield any information on the qualitative and quantitative chemical profiles. In order to focus not only on single quality marker compounds, more holistic concepts need to be developed such as metabolic fingerprinting [83,84]. Incorporating state-of-the-art analytical methodologies, including liquid chromatography / mass spectrometry (LC/MS), for chemical characterisation and chemical fingerprinting, differential cellular gene expression for bioresponse fingerprinting and animal pharmacology for *in-vivo* validation, PhytomicsQC, a proprietary comprehensive platform for quality control of botanical drugs was developed to ensure batch-to-batch consistency of a standardised TCM formula PHY906® [85]. The FP7 GP-TCM Consortium have recognised authentication and quality control of research materials used in clinical, animal and *in-vitro* studies as an area that needs urgent improvement and have developed a checklist and good practice guidelines [67,86]. Funding bodies, journals, academia and commercial suppliers must collaborate closely to address this fundamental issue. Post-harvest treatment and processing (*paozhi*) are common features in Chinese herbal medicine. In order to provide plant material of consistent quality, these processes need to be scientifically investigated and standard operating procedures and specific endpoints need to be defined and implemented [87].

#### Mechanisms and herbalome

Beyond the success in isolating new chemical entities amenable to drugs, such as artemisinin, emerging evidence suggested that multiple compounds from herbals might demonstrate synergism. *In vitro*, the antimicrobial action of berberine was potentiated by multidrug pump inhibitors biosynthesised by the same plants that produce berberine or by different plants [88,89]. In acute promyelocytic leukaemia (APL) animal models and in APL cell lines, synergism among three active compounds isolated from the three components of a TCM formula was also demonstrated [90]. In addition, Yung-Chi Cheng’s team at Yale University successfully combined animal studies, clinical studies and genomics technology to elucidate the mechanisms of PHY906® as a cancer adjuvant therapy and demonstrated that all four herbs in the PHY906® formulation were necessary for its biological effects [91-93]. In 2008, the “Herbalome Project”, an ambitious 15-year effort was launched in China to clarify the chemical composition, structure and function of commonly used Chinese herbs and TCM formulae, to establish a standard resource library, and to interpret the synergistic and complementary mechanisms of multiple components in TCM drugs on multiple targets [94,95]. Until recently, the project focused on development of systematic separation methodology for resolving and analysing the complex components in Chinese herbal medicine and establishment of a comprehensive resource library [95]. This project might eventually help to understand the chemical basis of the active constituents of TCM drugs, be they single or multiple compounds.

#### Acupuncture

Clinical trials on treatment of pain and other diseases, adverse effects, mechanisms of action and discussions on the roles for the placebo effect in acupuncture continued to be active. Earlier reports of adverse effects were usually related to insufficient basic medical knowledge, low hygienic standard and inadequate acupuncture education [96]. With disposable sterile needles and improved training and regulation of acupuncturists, more recent data from large surveys in the UK [97,98] and Germany [99,100] have shown that adverse events after acupuncture are uncommon and acupuncture is a safe therapy in the hands of well trained professionals. A placebo needle that does not penetrate the skin was introduced for sham acupuncture [35], a plausible tool to differentiate placebo effects caused by non-penetrating sham acupuncture [101,102]. A number of large-scale trials on the efficacy [103-105] and effectiveness [106,107] of acupuncture, as well as randomised controlled trials on the efficacy of combined therapy of acupuncture and Chinese herbal medicine [108], have been published; a recent meta-analysis, that pooled the data of 29 clinical trials on chronic pain, concluded that acupuncture is statistically superior to sham acupuncture [109]. However, debates about acupuncture point specificity, acupuncture techniques and placebo effects will likely continue. Nevertheless, a growing body of evidence had clinical implications. For example, in 2006, the German health authorities decided to include acupuncture in routine reimbursement by social health insurance funds for chronic low back pain and chronic osteoarthritis of the knee [110]; in 2009, the UK National Institute for Health and Clinical Excellence (NICE) issued a guidance on the treatment of low back pain, suggesting that doctors should ‘consider offering a course of acupuncture needling comprising a maximum of 10 sessions over a period of up to 12 weeks’ for their patients [111]; and in 2012, NICE published a new guideline on the diagnosis and management of headaches in young people and adults, which suggested that healthcare professionals should consider offering patients a course of up to 10 sessions of acupuncture, administered over a period of from five to eight weeks, for the prophylactic treatment of chronic tension-type headaches [112].

A number of physiological changes were documented as consequences of acupuncture needling, which might direct to possible mechanisms of action of acupuncture. In particular, a novel hypothesis for the physiological basis of acupuncture involving purinergic signalling was proposed, where adenosine-5’-triphosphate (ATP), released in the skin during acupuncture, acts on purinoceptors on sensory nerve terminals and, through interneurons, interrupts pain pathways and modulates motor nerve activity in the brain stem, regulating autonomic functions [113]. This was followed by a paper suggesting that adenosine, after breakdown of ATP in the skin, acted as a prejunctional modulator of nociceptive sensory pathways [114].

### The start of phase III (2011–2012) and the future

The start of Phase III coincided with the announcements of three prestigious international awards for scientists involved in different aspects of TCM research [115-117]:

• *The 2011 Lasker-DeBakey Clinical Medical Research Award* honoured Youyou Tu for the discovery of the anti-malaria drug artemisinin [15-17];

• *The 7*^*th*^*Annual Szent-Györgyi Prize* (2012) was awarded to Zhen-Yi Wang and Zhu Chen for their ground-breaking research of arsenic trioxide treatment of leukaemia back in the 1990s [20]; and

• *The 1*^*st*^*Cheung On Tak International Award for Outstanding Contribution to Chinese Medicine* (2012) was shared by Keji Chen for his pioneering research in TCM treatment of cardiovascular diseases [118,119] and to Yung-Chi Cheng for developing a classical TCM formula into a standardised modern cancer adjuvant therapy [93].

In October 2012, another highlight of the beginning of phase III was the final outputs from the FP7 GP-TCM project, which brought together a large collaborative network of over 200 scientists and clinicians, more than 100 institutions and 24 countries to work on the future directions of TCM. The main conclusions of this FP7 project were published in a special open-access issue of the *Journal of Ethnopharmacology*, in which good practice was defined, guidelines collated, information updated and priorities, challenges and opportunities agreed [120]. To sustainably develop, refine and disseminate good practices in TCM research beyond the FP7 GP-TCM project, the GP-TCM Research Association was founded in April 2012 [121].

The advice from the FP7 GP-TCM project to the TCM scientific community can be summarised by the need for more:

• Integrity: In the scientific context of TCM, integrity is defined as good practice and rigour, a concept of consistency of actions, values, methods, measures, principles, expectations and outcomes;

• Integration: The word refers to an approach towards wholeness by either bringing parts together or by removing barriers; and

• Innovation: The word refers to renewal or change, as well as introducing better or more effective products, processes, services, technologies, or ideas, etc.

These concepts are further expanded and interpreted below.

1. Integrity

• *Holism.* As a holistic medicine, TCM considers the human body as a whole, emphasises the importance of functions and emotions and considers patients as part of a system interacting with its environmental factors, such as diet, climate and life style. This is embodied in TCM diagnosis, prescriptions and life style interventions.

• *Ethics.* Since the era of Sun Simiao (581–682 A.D.) ethics has become an integral part of TCM [1]. In ancient China, practitioners’ ethics were at the centre of the TCM profession. Now, TCM ethics should apply to not only TCM practitioners, but also the agricultural, industrial and scientific communities, and all stakeholders. Conflict of interests should be avoided, stated and properly regulated.

• *Good practice*. As promulgated by the FP7 GP-TCM project and the GP-TCM Research Association, TCM requires continuous development, refinement and dissemination of good practice guidelines in all its multiple aspects. In such epic efforts, collaboration and sharing must be encouraged and we must strive for consensus while respecting differences. Good practice is especially required in authentication, quality control, safety assessments and sustainable use of TCM drugs and materials; agricultural and manufacturing practices; commercial and clinical practices; basic and clinical research of TCM; application of routine and emerging technologies; as well as differentiation between valuable knowledge and superstitious, erroneous and misleading anecdotes [120,121].

2. Integration

• *Education, clinical practice and research.* Integration of these three important aspects was one of the most important achievements in modernisation of TCM and this must continue.

• *Cultural, philosophical and scientific perspectives.* TCM is an important part of Chinese culture and is guided by Chinese philosophy. Thus, researchers of the scientific, cultural and philosophical values of TCM should collaborate with and learn from each other.

• *TCM, Western medicine and modern science*. In China and some other countries, TCM and Western medicine are both in mainstream healthcare and intend to complement each other. In the international context, dialogue between TCM and Western medicine needs to be promoted; China’s experiences and lessons should be studied in light of modern science so that both medical traditions may contribute to forging tomorrow’s medicine. Bringing evidence-based medicine to recognise the value of TCM will have to integrate a thorough rethinking of both Western and TCM practices to generate scientifically and statistically convincing evidence of the TCM-based approaches.

• *Interregional, intersectoral and interdisciplinary collaborations.* Due to the complexity and the vast range of TCM, collaborations between different regions, different business sectors, as well as different areas of knowledge must be encouraged in order to share resources and expertise and join forces to meet the challenges together. For example, the various distribution channels encountered in different countries must be connected in order to develop harmonised pharmacovigilance procedures suitable to rapidly and globally detect and assess warning signals for adverse events.

• *Holistic, relationist and analytical, reductionist approaches*. TCM emphasises holistic and relationist approaches of thinking, while Western medicine is largely based on analytical and reductionist approaches. To see both the trees and the wood, these approaches must be integrated.

• *Quality, toxicology and pharmacology*. These three most important aspects of TCM are so much interrelated that future training and research must further integrate these crucial elements to better ensure safety and efficacy.

3. Innovation

• *Modernisation of TCM is more than Westernisation*. Although some achievements have already arisen through studying TCM using a Western approach, e.g. isolating pure compounds, real innovations should include both TCM-inspired changes in mode of thinking and practice in Western medicine and TCM refinements inspired by modern science.

• *TCM diagnosis.* Among all aspects of TCM, holistic TCM diagnosis has probably the most complementary elements to modern medical practice, including its function-oriented description of organ systems and diagnostic approach (leading to syndrome differentiation), its emphasis on modulation of functional balance, its comprehensive categorisation and interpretation of tongue and pulse patterns, its characteristic categorisation of the nature of diseases and drugs, etc. These could be important sources for developing and validating innovative mindset, methods, tools and strategies that could complement biology-based diagnosis [122,123].

• *Preventive and comprehensive interventions.* TCM is characterised by both pre-emptive approaches and interventions with multiple components. It emphasises intervention before disease arises and often combines dietary advice, physical exercises such as Taijiquan (Tai Chi Chuan) [124,125], meditation, herbal medicines, massage, acupuncture and moxibustion, etc. The values of these individualised and integrated approaches are important directions for future public health.

• *Innovative, more robust theories and methodology.* The complexity of TCM demands novel and more robust ways of thinking, approaches, tools and methods. For example, the individualised and holistic nature of TCM requires tools for complexity research, research of the science of individuality and personalised medicine, as well as novel statistics [126]. It also awaits the maturation of omics, systems biology and other systems-based technologies [127].

• *Prioritisation and focus*. In view of the vast areas of TCM yet to be explored and the limited resources available for such an emerging area of research in the global context, prioritisation and focus become the key to achieving real innovation. In this regard, a Steve Jobs approach for innovation is to **“say no to 1,000 things”**[128] and to focus only on those that could make real differences becomes all the more compelling.

## Conclusions

After 60 years of development, modernisation of TCM has arrived at a new era. Accumulating evidence supports the notion that this new era represents a golden opportunity for TCM to further consolidate its scientific base so as to play a bigger part in forging tomorrow’s medicines. To achieve this, especially to reach the goals of better quality, safety and efficacy, the proposed rules of *integrity*, *integration* and *innovation* must be followed.

## Abbreviations

APL: Acute promyelocytic leukaemia; ATP: Adenosine-5’-triphosphate; EMA: The European Medicines Agency; EU: European Union; FDA: The Food and Drug Administration of the United States; FP7: The 7th Framework Programme; GAP: Good Agricultural Practice; GDP: Gross domestic product; GP-TCM: Good Practice in Traditional Chinese Medicine; HEIs: Higher education institutions; HMPC: The Committee on Herbal Medicinal Products; IND: Investigational new drug; ISO: International Organization of Standardization; LC: Liquid chromatography; MS: Mass spectrometry; NCCAM: The National Center for Complementary and Alternative Medicine of the United States; NICE: The UK National Institute for Health and Clinical Excellence; NICM: Australian Natural Institute of Complementary Medicine; R&D: Research and development; SATCM: State Administration of Traditional Chinese Medicine of China; TCM: Traditional Chinese medicine; UNESCO: The United Nations Educational, Scientific and Cultural Organisation; UK: United Kingdom; USA: United States of America; WHO: The World Health Organization

## Competing interest

The authors declare that they have no competing interests.

## Authors’ contributions

QX contributed to the overall conceptualisation and project plan, coordinated various sections of the paper, synthesised the findings and coordinated the writing of the paper. RB contributed to the overall writing and revision, especially on milestones outside China and in sections on TCM resources, quality control and standardisation. PJH and BMH contributed to the overall conceptualisation, synthesised the findings and played central roles in discussion and revision of the manuscript. T-PF contributed to the overall revision and provided some very critical references and ideas. ZZ provided the photo used in Figure 1 and contributed to the writing of the TCM resources section. PD contributed to the writing and critical revision of the whole manuscript, especially the toxicology section. CMW and NR gathered literature and information and wrote part of the acupuncture section, as well as contributed to the overall discussion and revision process. MSJS did literature analysis for the whole paper and contributed to the overall discussion and revision of the manuscript. AL provided information on clinical TCM and integrative TCM studies and wrote part of the contents of the paper. DG provided some useful discussions and revisions on the manuscript. All authors read and approved the final manuscript.

## Authors’ information

QX is Senior Lecturer in Renal Medicine and Co-Director of the King’s Centre for Integrative Chinese Medicine at King’s College London. He was the Coordinator of the FP7 GP-TCM Project and is Vice President of the GP-TCM Research Association. RB is Professor of Pharmacognosy, Head of the Institute of Pharmaceutical Sciences at University of Graz, Head of TCM Research Center Graz, member of the European Pharmacopoeia TCM Working Party, and President of the GP-TCM Research Association. BMH is Professor of Renal Medicine at King’s College London and President-elect of the Renal Association, UK. T-PF is Senior Lecturer and Head of Angiogenesis and Chinese Medicine Laboratory in the Department of Pharmacology, University of Cambridge. He was Deputy Coordinator of the FP7 GP-TCM Project and Coordinator of its R&D and Regulatory Work Package, and is Secretary-General of the GP-TCM Research Association. AL and ZZ, Professors of TCM, are respectively Dean and Associate Dean of School of Chinese Medicine, the Hong Kong Baptist University. PD is Professor of Pharmacognosy at Université Libre de Bruxelles and Université de Mons, member of the European Pharmacopoeia TCM Working Party, and was Co-Coordinator of the Toxicology Work Package of the FP7 GP-TCM Project. CMW is Professor of Medicine and Acting Director of Institute for Social Medicine, Epidemiology and Health Economics at Charité-Universitätsmedizin Berlin. NR is Professor of TCM and Integrated Health at the London South Bank University and was Co-Coordinator of the Clinical Studies of Chinese Herbal Medicine and Acupuncture Work Packages of the FP7 GP-TCM Project. MSJS is Director of the Kew Innovation Unit, Deputy Keeper & Head of Sustainable Uses of Plants Group at Royal Botanic Gardens, Kew, and was Coordinator of the Quality Control Work Package of the FP7 GP-TCM Project. DG is Chair Professor of Pharmacognosy and Director of Center for TCM Modernization, Shanghai Institute of Materia Medica, Chinese Academy of Sciences. He was Deputy Coordinator of the FP7 GP-TCM Project and is President-elect of the GP-TCM Research Association, and chairs the Natural Medicine Expert Committee of the Chinese Pharmacopoeia and sits on the Expert Committee of the United States Pharmacopeia. PJH is Professor of Pharmaceutical Chemistry, Director of the Institute of Pharmaceutical Science and Co-Director of the King’s Centre for Integrative Chinese Medicine at King’s College London. He was Coordinator of the *In-Vitro* Pharmacology Work Package and chaired the Technological Advisory Board of the FP7 GP-TCM Project and is a member of the Expert Advisory Group on Herbal Medicinal Products, British Pharmacopoeia Commission. QX, RB, T-PF, PD, MSJS, AL, NR, DG and PJH are members of the Board of Directors of the GP-TCM Research Association.

## Pre-publication history

The pre-publication history for this paper can be accessed here:

http://www.biomedcentral.com/1472-6882/13/132/prepub

## References

[B1] Cai J Integration of traditional Chinese medicine with Western medicine – Right or wrong Soc Sci Med 1988 27 521 529 10.1016/0277-9536(88)90376-0 3227361

[B2] Fazel M Now show me your tongue: a taste of medicine in China Lancet 1995 346 1687 1688 10.1016/S0140-6736(95)92847-2 8551831

[B3] Scheid V The globalization of Chinese medicine Lancet 1999 354 SIV10 10691420 10.1016/s0140-6736(99)90353-7

[B4] Tang JL Liu BY Ma KW Traditional Chinese medicine Lancet 2008 372 1938 1940 10.1016/S0140-6736(08)61354-9 18930523

[B5] Unschuld PU The past 1000 years of Chinese medicine Lancet 1999 354 SIV9 10691484 10.1016/s0140-6736(99)90352-5

[B6] Qiu J China plans to modernize traditional medicine Nature 2007 446 590 591 10.1038/446590a 17410143

[B7] Wright P Passport to Peking: A very British mission to Mao's China 2010 Oxford: Oxford University Press

[B8] James DW Chinese medicine Lancet 1955 265 1068 1069 14368962 10.1016/s0140-6736(55)91135-1

[B9] Porkert M Klinische chinesische Pharmakologie 1978 Heidelberg: Verlag für Medizin Fischer

[B10] Harmsworth K Lewith GT Attitudes to traditional Chinese medicine amongst Western trained doctors in the People's Republic of China Soc Sci Med 2001 52 149 153 10.1016/S0277-9536(00)00124-6 11144912

[B11] Gaw AC Chang LW Shaw L-C Efficacy of acupuncture on osteoarthritic pain. A controlled, double-blind study N Engl J Med 1975 293 375 378 10.1056/NEJM197508212930803 1097921

[B12] Pomeranz B Chiu D Naloxone blockade of acupuncture analgesia: endorphin implicated Life Sci 1976 19 1757 1762 10.1016/0024-3205(76)90084-9 187888

[B13] Mayer DJ Price DD Rafii A Antagonism of acupuncture analgesia in man by the narcotic antagonist naloxone Brain Res 1977 121 368 372 10.1016/0006-8993(77)90161-5 832169

[B14] Han JS Acupuncture and endorphins Neurosci Lett 2004 361 258 261 10.1016/j.neulet.2003.12.019 15135942

[B15] Tu Y The discovery of artemisinin (qinghaosu) and gifts from Chinese medicine Nat Med 2011 17 1217 1220 10.1038/nm.2471 21989013

[B16] Miller LH Su X Artemisinin: discovery from the Chinese herbal garden Cell 2011 146 855 858 10.1016/j.cell.2011.08.024 21907397 PMC3414217

[B17] Neill US From branch to bedside: Youyou Tu is awarded the 2011 Lasker~DeBakey Clinical Medical Research Award for discovering artemisinin as a treatment for malaria J Clin Invest 2011 121 3768 3773 10.1172/JCI60887 22059236 PMC3195493

[B18] Wall ME Wani MC Cook CE Palmer KH McPhail AT Sim GA Plant antitumor agents. I. The isolation and structure of camptothecin, a novel alkaloidal leukemia and tumor inhibitor from Camptotheca acuminata J Am Chem Soc 1966 88 3888 3890 10.1021/ja00968a057

[B19] Wall M Wani M Camptothecin: Discovery to Clinic Ann N Y Acad Sci 1996 803 1 12 10.1111/j.1749-6632.1996.tb26371.x 8993495

[B20] Shen ZX Chen GQ Ni JH Li XS Xiong SM Qiu QY Zhu J Tang W Sun GL Yang KQ Chen Y Zhou L Fang ZW Wang YT Ma J Zhang P Zhang TD Chen SJ Chen Z Wang ZY Use of arsenic trioxide (As_2_O_3_) in the treatment of acute promyelocytic leukemia (APL): II. Clinical efficacy and pharmacokinetics in relapsed patients Blood 1997 89 3354 3360 9129042

[B21] Wall ME Wani MC Camptothecin and taxol: discovery to clinic - Thirteenth Bruce F. Cain Memorial Award Lecture Cancer Res 1995 55 753 760 7850785

[B22] Melchart D Linde K Weidenhammer W Hager S Liao J Bauer R Wagner H Use of traditional drugs in a hospital of Chinese medicine in Germany Pharmacoepidemiol Drug Saf 1999 8 115 120 10.1002/(SICI)1099-1557(199903/04)8:2<115::AID-PDS412>3.0.CO;2-I 15073936

[B23] Wang S On the modalities of modern traditional Chinese medicine education Journal of Shanxi College of Traditional Chinese Medicine 2012 3 69 71

[B24] Pearson NJ Chesney MA The National Center for Complementary and Alternative Medicine Acad Med 2007 82 967 10.1097/ACM.0b013e31814a5462 17895659

[B25] The Australian National Institute of Complementary Medicine websitehttp://www.nicm.edu.au/about-nicm

[B26] Medicine and Health The Eurasia-Pacific Uninet webpagehttp://www.eurasiapacific.net/index.php?page=content&pid=18

[B27] Uzuner H Fan TP Dias A Guo DA El-Nezami HS Xu Q Establishing an EU-China consortium on traditional Chinese medicine research Chin Med 2010 5 42 10.1186/1749-8546-5-42 21156056 PMC3019128

[B28] World Health Organization webpage for the article Medicinal Plants in China A Selection of 150 Commonly Used Specieshttp://whqlibdoc.who.int/wpro/-1993/9290611022.pdf

[B29] Burr W WHO moves to classify traditional medicines CMAJ 2011 183 E73 E74 21173064 10.1503/cmaj.109-3761 PMC3033939

[B30] ISO webpage for its TCM Technical Committeehttp://www.iso.org/iso/standards_development/technical_committees/other_bodies/iso_technical_committee.htm?commid=598435

[B31] UNESCO web page on acupuncture and moxibustion http://www.unesco.org/culture/ich/en/RL/00425

[B32] UNESCO web page on Ben Cao Gang Mu (Compendium of Materia Medica) http://www.unesco.org/new/en/communication-and-information/flagship-project-activities/memory-of-the-world/register/full-list-of-registered-heritage/registered-heritage-page-1/ben-cao-gang-mu-compendium-of-materia-medica/

[B33] UNESCO web page on Huang Di Nei Jing (Yellow Emperor's Inner Canon) http://www.unesco.org/new/en/communication-and-information/flagship-project-activities/memory-of-the-world/register/full-list-of-registered-heritage/registered-heritage-page-4/huang-di-nei-jing-yellow-emperors-inner-canon/15555247

[B34] Skrabanek P Acupuncture and the age of unreason Lancet 1984 323 1169 1171 10.1016/S0140-6736(84)91406-5 6144887

[B35] Streitberger K Kleinhenz J Introducing a placebo needle into acupuncture research Lancet 1998 352 364 365 10.1016/S0140-6736(97)10471-8 9717924

[B36] Witt C Brinkhaus B Jena S Linde K Streng A Wagenpfeil S Hummelsberger J Walther HU Melchart D Willich SN Acupuncture in patients with osteoarthritis of the knee: a randomised trial Lancet 2005 366 136 143 10.1016/S0140-6736(05)66871-7 16005336

[B37] Cheng TO Medicine in China Lancet 1996 347 774 10.1016/S0140-6736(96)90135-X 8602047

[B38] Fugh-Berman A Herb-drug interactions Lancet 2000 355 134 138 10.1016/S0140-6736(99)06457-0 10675182

[B39] Fushimi R Tachi J Amino N Miyai K Chinese medicine interfering with digoxin immunoassays Lancet 1989 333 339 2563502 10.1016/s0140-6736(89)91361-5

[B40] Atherton DJ Sheehan MP Rustin M Traditional Chinese plants for eczema Lancet 1991 338 510 1678465 10.1016/0140-6736(91)90580-i

[B41] Sheehan MP Rustin MH Atherton DJ Buckley C Harris DW Brostoff J Ostlere L Dawson A Efficacy of traditional Chinese herbal therapy in adult atopic dermatitis Lancet 1992 340 13 17 10.1016/0140-6736(92)92424-E 1351600

[B42] Chan TY Chan JC Tomlinson B Critchley JA Chinese herbal medicines revisited: a Hong Kong perspective Lancet 1993 342 1532 1534 10.1016/S0140-6736(05)80091-1 7902907

[B43] Jadoul M de Plaen JF Cosyns JP Van Ypersele de Strihou C Adverse effects from traditional Chinese medicine Lancet 1993 341 892 893 10.1016/0140-6736(93)93099-M 8096589

[B44] Tai YT But PP Young K Lau CP Adverse effects from traditional Chinese medicine Lancet 1993 341 892 10.1016/0140-6736(93)93099-M 8096588

[B45] Hanson M Maoist public-health campaigns, Chinese medicine, and SARS Lancet 2008 372 1457 1458 10.1016/S0140-6736(08)61610-4 18975392 PMC7137983

[B46] Lampert N Complementary and alternative medicine Lancet 2001 357 802 11253994 10.1016/S0140-6736(05)71226-5

[B47] Ross C New life for old medicine Lancet 1993 342 485 486 8102437 10.1016/0140-6736(93)91602-i

[B48] Okada F Kampo medicine, a source of drugs waiting to be exploited Lancet 1996 348 5 6 8691936 10.1016/s0140-6736(05)64351-6

[B49] Wang J Evidence-based medicine in China Lancet 2010 375 532 533 10.1016/S0140-6736(09)62131-0 20159275

[B50] Sheng-Ji P Ethnobotanical approaches of traditional medicine studies: some experiences from Asia Pharm Biol 2001 39 Suppl 1 74 79 21554174 10.1076/phbi.39.s1.74.0005

[B51] Xiao PG Pictorial of Encyclopaedia of Chinese Herbal Medicine 1988-1997 1–12 Hong Kong: The Commercial Press

[B52] Li D Xiao P Liu C Modern Research and Application of Chinese Medicinal Plants 2000 Hong Kong: Hong Kong Medical Publisher

[B53] Xiao PG Modern Chinese Materia Medica 2002–2007 1–5 Beijing: Chemical Industry Press

[B54] Zhao ZZ Xiao PG Encyclopedia of Contemporary Medicinal Plants (English Version) 2010 1–4 Shanghai: World Publishing Corporation

[B55] Wagner H Bauer R Melchart D Xiao PG Staudinger A Chromatographic Fingerprint Analysis of Herbal Medicines - Thin-layer and High Performance Liquid Chromatography of Chinese Drugs 2011 1- 2 Vienna: Springer-Verlag

[B56] Bauer R Franz G Modern European monographs for quality control of Chinese herbs Planta Med 2010 76 2004 2011 10.1055/s-0030-1250532 21077026

[B57] Sheehan MP Atherton DJ One-year follow up of children treated with Chinese medicinal herbs for atopic eczema Br J Dermatol 1994 130 488 493 10.1111/j.1365-2133.1994.tb03383.x 8186115

[B58] Batey RG Bensoussan A Fan YY Bollipo S Hossain MA Preliminary report of a randomized, double-blind placebo-controlled trial of a Chinese herbal medicine preparation CH-100 in the treatment of chronic hepatitis C J Gastroenterol Hepatol 1998 13 244 247 10.1111/j.1440-1746.1998.01550.x 9570235

[B59] Bensoussan A Talley NJ Hing M Menzies R Guo A Ngu M Treatment of irritable bowel syndrome with Chinese herbal medicine: a randomized controlled trial JAMA 1998 280 1585 1589 10.1001/jama.280.18.1585 9820260

[B60] Normile D The New Face of Traditional Chinese Medicine Science 2003 299 188 190 10.1126/science.299.5604.188 12522228

[B61] Fan TP Deal G Koo HL Rees D Sun H Chen S Dou JH Makarov VG Pozharitskaya ON Shikov AN Kim YS Huang YT Chang YS Jia W Dias A Wong VC Chan K Future development of global regulations of Chinese herbal products J Ethnopharmacol 2012 140 568 586 10.1016/j.jep.2012.02.029 22373513

[B62] Jiang M Yang J Zhang C Liu B Chan K Cao H Lu A Clinical studies with traditional Chinese medicine in the past decade and future research and development Planta Med 2010 76 2048 2064 10.1055/s-0030-1250456 20979016

[B63] Junhua Z Hongcai S Xiumei G Boli Z Yaozu X Hongbo C Ming R Methodology and reporting quality of systematic review/meta-analysis of traditional Chinese medicine J Altern Complement Med 2007 13 797 805 10.1089/acm.2007.7195 17983335

[B64] He Y Lu A Zha Y Yan X Song Y Zeng S Liu W Zhu W Su L Feng X Qian X Lu C Correlations between symptoms as assessed in traditional chinese medicine (TCM) and ACR20 efficacy response: a comparison study in 396 patients with rheumatoid arthritis treated with TCM or Western medicine J Clin Rheumatol 2007 13 317 321 10.1097/RHU.0b013e31815d019b 18176139

[B65] Wang C Cao B Liu QQ Zou ZQ Liang ZA Gu L Dong JP Liang LR Li XW Hu K He XS Sun YH An Y Yang T Cao ZX Guo YM Wen XM Wang YG Liu YL Jiang LD Oseltamivir compared with the Chinese traditional therapy maxingshigan-yinqiaosan in the treatment of H1N1 influenza: a randomized trial Ann Intern Med 2011 155 217 225 10.7326/0003-4819-155-4-201108160-00005 21844547

[B66] Flower A Witt C Liu JP Ulrich-Merzenich G Yu H Lewith G Guidelines for randomised controlled trials investigating Chinese herbal medicine J Ethnopharmacol 2012 140 550 554 10.1016/j.jep.2011.12.017 22210103

[B67] Chan K Shaw D Simmonds MS Leon CJ Xu Q Lu A Sutherland I Ignatova S Zhu YP Verpoorte R Williamson EM Duez P Good practice in reviewing and publishing studies on herbal medicine, with special emphasis on traditional Chinese medicine and Chinese materia medica J Ethnopharmacol 2012 140 469 475 10.1016/j.jep.2012.01.038 22330011

[B68] Ouedraogo M Baudoux T Stévigny C Nortier J Colet JM Efferth T Qu F Zhou J Chan K Shaw D Pelkonen O Duez P Review of current and “omics” methods for assessing the toxicity (genotoxicity, teratogenicity and nephrotoxicity) of herbal medicines J Ethnopharmacol 2012 140 492 512 10.1016/j.jep.2012.01.059 22386524

[B69] De Broe ME Chinese herbs nephropathy and Balkan endemic nephropathy: toward a single entity, aristolochic acid nephropathy Kidney Int 2012 81 513 515 10.1038/ki.2011.428 22373701

[B70] Chen CH Dickman KG Moriya M Zavadil J Sidorenko VS Edwards KL Gnatenko DV Wu L Turesky RJ Wu XR Pu YS Grollman AP Aristolochic acid-associated urothelial cancer in Taiwan Proc Natl Acad Sci USA 2012 109 8241 8246 10.1073/pnas.1119920109 22493262 PMC3361449

[B71] The booklet Towards a Safer Choice- The Practice of Chinese Medicine In Australia by Bensoussan A & Myers SP on the website of Department of Human Services, Victoria, Australiahttp://www.health.vic.gov.au/archive/archive2006/chinese/report/contents.html

[B72] Fu PP Xia QS Lin G Chou MW Pyrrolizidine alkaloids-genotoxicity, metabolism enzymes, metabolic activation, and mechanisms Drug Metab Rev 2004 36 1 55 15072438 10.1081/dmr-120028426

[B73] Shaw D Graeme L Pierre D Elizabeth W Kelvin C Pharmacovigilance of herbal medicine J Ethnopharmacol 2012 140 513 518 10.1016/j.jep.2012.01.051 22342381

[B74] Zhang L Yan J Liu X Ye Z Yang X Meyboom R Chan K Shaw D Duez P Pharmacovigilance practice and risk control of Traditional Chinese Medicine drugs in China: current status and future perspective J Ethnopharmacol 2012 140 519 525 10.1016/j.jep.2012.01.058 22374080

[B75] Chen ST Dou J Temple R Agarwal R Wu KM Walker S New therapies from old medicines Nat Biotechnol 2008 26 1077 1083 10.1038/nbt1008-1077 18846070

[B76] Vlietinck A Pieters L Apers S Legal requirements for the quality of herbal substances and herbal preparations for the manufacturing of herbal medicinal products in the European Union Planta Med 2009 75 683 688 10.1055/s-0029-1185307 19204891

[B77] Dobos GJ Tan L Cohen MH McIntyre M Bauer R Li X Bensoussan A Are national quality standards for traditional Chinese herbal medicine sufficient? Current governmental regulations for traditional Chinese herbal medicine in certain Western countries and China as the Eastern origin country Compl Ther Med 2005 13 183 190 10.1016/j.ctim.2005.06.004 16150372

[B78] Zhang B Peng Y Zhang Z Liu H Qi Y Liu S Xiao P GAP production of TCM herbs in China Planta Med 2010 76 1948 1955 10.1055/s-0030-1250527 21077024

[B79] Heuberger H Bauer R Friedl F Heubl G Hummelsberger J Nögel R Seidenberger R Torres-Londoño P Cultivation and breeding of Chinese medicinal plants in Germany Planta Med 2010 76 1956 1962 10.1055/s-0030-1250528 21077027

[B80] Zhao Z Guo P Brand E The formation of daodi medicinal materials J Ethnopharmacol 2012 140 476 481 10.1016/j.jep.2012.01.048 22342382

[B81] Pferschy-Wenzig E-M Bauer R Houghton P, Mukherjee PK Quality control of Chinese herbal drugs Evaluation of herbal medicinal products: perspectives on quality, safety, and efficacy 2009 London: Pharmaceutical Press 1 502

[B82] Heubl G New aspects of DNA-based authentication of Chinese medicinal plants by molecular biological techniques Planta Med 2010 76 1963 1974 10.1055/s-0030-1250519 21058240

[B83] Liang YZ Xie PS Chan K Perspective of chemical fingerprinting of Chinese herbs Planta Med 2010 76 1997 2003 10.1055/s-0030-1250541 21064007

[B84] Sheridan H Krenn L Jiang R Sutherland I Ignatova S Marmann A Liang X Sendker J The potential of metabolic fingerprinting as a tool for the modernisation of TCM preparations J Ethnopharmacol 2012 140 482 491 10.1016/j.jep.2012.01.050 22338647

[B85] Tilton R Paiva AA Guan JQ Marathe R Jiang Z van Eyndhoven W Bjoraker J Prusoff Z Wang H Liu SH Cheng YC A comprehensive platform for quality control of botanical drugs (PhytomicsQC): a case study of Huangqin Tang (HQT) and PHY906 Chin Med 2010 5 30 10.1186/1749-8546-5-30 20727161 PMC2940884

[B86] Tejedor Garcia N Garcia Bermejo L Fernandez Martinez AB Olmos Centenera G Kumari R Xu Q Cheng X Watson S de Lucio Cazaña FJ MEDLINE-based assessment of animal studies on Chinese herbal medicine J Ethnopharmacol 2012 140 545 549 10.1016/j.jep.2012.02.008 22353429

[B87] Zhao ZZ Liang ZT Chan K Lu GH Lee ELM Chen HB Li L A unique issue in the standardization of Chinese Materia Medica: Processing Planta Med 2010 76 1975 1986 10.1055/s-0030-1250522 21049396

[B88] Stermitz FR Lorenz P Tawara JN Zenewicz LA Lewis K Synergy in a medicinal plant: antimicrobial action of berberine potentiated by 5’-methoxyhydnocarpin, a multidrug pump inhibitor Proc Natl Acad Sci USA 2000 97 1433 1437 10.1073/pnas.030540597 10677479 PMC26451

[B89] Junio HA Sy-Cordero AA Ettefagh KA Burns JT Micko KT Graf TN Richter SJ Cannon RE Oberlies NH Cech NB Synergy-directed fractionation of botanical medicines: a case study with goldenseal (Hydrastis canadensis) J Nat Prod 2011 74 1621 1629 10.1021/np200336g 21661731 PMC3142294

[B90] Wang L Zhou GB Liu P Song JH Liang Y Yan XJ Xu F Wang BS Mao JH Shen ZX Chen SJ Chen Z Dissection of mechanisms of Chinese medicinal formula Realgar-Indigo naturalis as an effective treatment for promyelocytic leukemia Proc Natl Acad Sci USA 2008 105 4826 4831 10.1073/pnas.0712365105 18344322 PMC2290784

[B91] Lam W Bussom S Guan F Jiang Z Zhang W Gullen EA Liu SH Cheng YC The four-herb Chinese medicine PHY906 reduces chemotherapy-induced gastrointestinal toxicity Sci Transl Med 2010 2 45ra59 10.1126/scitranslmed.3001270 20720216

[B92] Wang E Bussom S Chen J Quinn C Bedognetti D Lam W Guan F Jiang Z Mark Y Zhao Y Stroncek DF White J Marincola FM Cheng YC Interaction of a traditional Chinese Medicine (PHY906) and CPT-11 on the inflammatory process in the tumor microenvironment BMC Med Genomics 2011 4 38 10.1186/1755-8794-4-38 21569348 PMC3117677

[B93] Liu SH Cheng YC Old formula, new Rx: the journey of PHY906 as cancer adjuvant therapy J Ethnopharmacol 2012 140 614 623 10.1016/j.jep.2012.01.047 22326673

[B94] Stone R Biochemistry. Lifting the veil on traditional Chinese medicine Science 2008 319 709 710 10.1126/science.319.5864.709 18258866

[B95] Zhang X Liu Y Guo Z Feng J Dong J Fu Q Wang C Xue X Xiao Y Liang X The herbalome - an attempt to globalize Chinese herbal medicine Anal Bioanal Chem 2012 402 573 581 10.1007/s00216-011-5533-y 22089819

[B96] Norheim AJ Adverse effects of acupuncture: a study of the literature for the years 1981–1994 J Altern Complement Med 1996 2 291 297 10.1089/acm.1996.2.291 9395661

[B97] MacPherson H Thomas K Walters S Fitter M The York acupuncture safety study: prospective survey of 34,000 treatments by traditional acupuncturists BMJ 2001 323 486 487 10.1136/bmj.323.7311.486 11532841 PMC48134

[B98] White A Hayhoe S Hart A Ernst E Adverse events following acupuncture: prospective survey of 32,000 consultations with doctors and physiotherapists BMJ 2001 323 485 486 10.1136/bmj.323.7311.485 11532840 PMC48133

[B99] Melchart D Weidenhammer W Streng A Reitmayr S Hoppe A Ernst E Linde K Prospective investigation of adverse effects of acupuncture in 97733 patients Arch Intern Med 2004 164 104 105 10.1001/archinte.164.1.104 14718331

[B100] Witt CM Pach D Brinkhaus B Wruck K Tag B Mank S Willich SN Safety of acupuncture: results of a prospective observational study with 229, 230 patients and introduction of a medical information and consent form Forsch Komplementarmed 2009 16 91 97 10.1159/000209315 19420954

[B101] Linde K Witt CM Streng A Weidenhammer W Wagenpfeil S Brinkhaus B Willich SN Melchart D The impact of patient expectations on outcomes in four randomized controlled trials of acupuncture in patients with chronic pain Pain 2007 128 264 271 10.1016/j.pain.2006.12.006 17257756

[B102] Wechsler ME Kelley JM Boyd IO Dutile S Marigowda G Kirsch I Israel E Kaptchuk TJ Active albuterol or placebo, sham acupuncture, or no intervention in asthma N Engl J Med 2011 365 119 126 10.1056/NEJMoa1103319 21751905 PMC3154208

[B103] Berman BM Lao L Langenberg P Lee WL Gilpin AM Hochberg MC Effectiveness of acupuncture as adjunctive therapy in osteoarthritis of the knee: a randomized, controlled trial Ann Intern Med 2004 141 901 910 10.7326/0003-4819-141-12-200412210-00006 15611487

[B104] Haake M Müller HH Schade-Brittinger C Basler HD Schäfer H Maier C Endres HG Trampisch HJ Molsberger A German Acupuncture Trials (GERAC) for chronic low back pain: randomized, multicenter, blinded, parallel-group trial with 3 groups Arch Intern Med 2007 167 1892 1898 10.1001/Archinte.167.17.1892 17893311

[B105] Linde K Streng A Jürgens S Hoppe A Brinkhaus B Witt C Wagenpfeil S Pfaffenrath V Hammes MG Weidenhammer W Willich SN Melchart D Acupuncture for patients with migraine: a randomized controlled trial JAMA 2005 293 2118 2125 10.1001/jama.293.17.2118 15870415

[B106] Vickers AJ Rees RW Zollman CE McCarney R Smith CM Ellis N Fisher P Van Haselen R Acupuncture for chronic headache in primary care: large, pragmatic, randomised trial BMJ 2004 328 744 10.1136/bmj.38029.421863.EB 15023828 PMC381326

[B107] Witt CM Jena S Selim D Brinkhaus B Reinhold T Wruck K Liecker B Linde K Wegscheider K Willich SN Pragmatic randomized trial evaluating the clinical and economic effectiveness of acupuncture for chronic low back pain Am J Epidemiol 2006 164 487 496 10.1093/aje/kwj224 16798792

[B108] Brinkhaus B Hummelsberger J Kohnen R Seufert J Hempen CH Leonhardy H Nögel R Joos S Hahn E Schuppan D Acupuncture and Chinese herbal medicine in the treatment of patients with seasonal allergic rhinitis: a randomized- controlled clinical trial Allergy 2004 59 953 960 10.1111/j.1398-9995.2004.00540.x 15291903

[B109] Vickers AJ Cronin AM Maschino AC Lewith G Macpherson H Foster NE Sherman KJ Witt CM Linde K for the Acupuncture Trialists’ Collaboration: **Acupuncture for chronic pain: individual patient meta-analysis** Arch Intern Med 2012 172 1444 1453 22965186 10.1001/archinternmed.2012.3654 PMC3658605

[B110] Cummings M Modellvorhaben Akupunktur - a summary of the ART, ARC and GERAC trials Acupunct Med 2009 27 26 30 10.1136/aim.2008.000281 19369191

[B111] Guidance on the management of back pain on the UK National Institute for Health and Clinical Excellence websitehttp://www.nice.org.uk/nicemedia/live/11887/44343/44343.pdf

[B112] Guidance on the diagnosis and management of headaches on the UK National Institute for Health and Clinical Excellence websitehttp://www.nice.org.uk/nicemedia/live/13901/60853/60853.pdf

[B113] Burnstock G Acupuncture: a novel hypothesis for the involvement of purinergic signalling Med Hypotheses 2009 73 470 472 10.1016/j.mehy.2009.05.031 19628336

[B114] Goldman N Chen M Fujita T Xu Q Peng W Liu W Jensen TK Pei Y Wang F Han X Chen JF Schnermann J Takano T Bekar L Tieu K Nedergaard M Adenosine A1 receptors mediate local anti-nociceptive effects of acupuncture Nat Neurosci 2010 13 883 888 10.1038/nn.2562 20512135 PMC3467968

[B115] Lasker~DeBakey Clinical Medical Research Award 2011 Winnershttp://www.laskerfoundation.org/awards/2011_c_description.htm

[B116] National Foundation for Cancer Research Szent-Györgyi Prizehttp://www.nfcr.org/asg-prize/prize-winners

[B117] Cheung On Tak International Award for Outstanding Contribution to Chinese Medicinehttp://scm.hkbu.edu.hk/en/cm-award/intro/index.html

[B118] Chen K Zhang Z Liang Z Blood stasis and research of activating blood circulation and eliminating stasis 1990 Shanghai: Shanghai Science & Technology Press

[B119] Liu Y Yin HJ Shi DZ Chen KJ Chinese herb and formulas for promoting blood circulation and removing blood stasis and antiplatelet therapies Evid Based Complement Alternat Med 2012 201 2 184503 22454656 10.1155/2012/184503 PMC3292253

[B120] Uzuner H Bauer R Fan TP Guo DA Dias A El-Nezami H Efferth T Williamson EM Heinrich M Robinson N Hylands PJ Hendry BM Cheng YC Xu Q Traditional Chinese medicine research in the post-genomic era: Good practice, priorities, challenges and opportunities J Ethnopharmacol 2012 140 458 468 10.1016/j.jep.2012.02.028 22387462

[B121] The GP-TCM Research Association websitehttp://www.gp-tcm.org

[B122] Ehrman TM Barlow DJ Hylands PJ Phytochemical informatics of traditional Chinese medicine and therapeutic relevance J Chem Inf Model 2007 47 2316 2334 10.1021/ci700155t 17929800

[B123] van der Greef J Perspective: All systems go Nature 2011 480 S87 10.1038/480S87a 22190087

[B124] Wang C Schmid CH Rones R Kalish R Yinh J Goldenberg DL Lee Y McAlindon T A randomized trial of tai chi for fibromyalgia N Engl J Med 2010 363 743 754 10.1056/NEJMoa0912611 20818876 PMC3023168

[B125] Li F Harmer P Fitzgerald K Eckstrom E Stock R Galver J Maddalozzo G Batya SS Tai chi and postural stability in patients with Parkinson's disease N Engl J Med 2012 366 511 519 10.1056/NEJMoa1107911 22316445 PMC3285459

[B126] van der Greef J Hankemeier T McBurney RN Metabolomics-based systems biology and personalized medicine: moving towards n = 1 clinical trials? Pharmacogenomics 2006 7 1087 1094 10.2217/14622416.7.7.1087 17054418

[B127] Buriani A Garcia-Bermejo ML Bosisio E Xu Q Li H Dong X Simmonds MS Carrara M Tejedor N Lucio-Cazana J Hylands PJ Omic techniques in systems biology approaches to traditional Chinese medicine research: present and future J Ethnopharmacol 2012 140 535 544 10.1016/j.jep.2012.01.055 22342380

[B128] Gallo C The innovation secrets of Steve Jobs 2011 Columbus: McGraw-Hill Companies, Inc

